# *Aronia melanocarpa* Fruit Juice Modulates ACE2 Immunoexpression and Diminishes Age-Related Remodeling of Coronary Arteries in Rats

**DOI:** 10.3390/foods11091220

**Published:** 2022-04-23

**Authors:** Elena Daskalova, Slavi Delchev, Lyudmila Vladimirova-Kitova, Iliya Bivolarski, Mina Pencheva, Petko Denev

**Affiliations:** 1 Department of Anatomy, Histology and Embryology, Medical Faculty, Medical University of Plovdiv, 4002 Plovdiv, Bulgaria; eli_das@abv.bg (E.D.); sldel@abv.bg (S.D.); 2 First Department of Internal Diseases—Section of Cardiology, Medical Faculty, Medical University of Plovdiv, 4002 Plovdiv, Bulgaria; kitov@vip.bg; 3 Department of General and Clinical Pathology, Medical Faculty, Medical University of Plovdiv, 4002 Plovdiv, Bulgaria; iliya_bivolarski@abv.bg; 4 Department of Medical Physics and Biophysics, Faculty of Pharmacy, Medical University of Plovdiv, 4002 Plovdiv, Bulgaria; minapencheva@ymail.com; 5 Laboratory of Biologically Active Substances, Institute of Organic Chemistry with Centre of Phytochemistry, Bulgarian Academy of Science, 4000 Plovdiv, Bulgaria

**Keywords:** ageing arteries, black chokeberry (*Aronia melanocarpa*), ACE2, functional foods

## Abstract

The aim of the study is to evaluate the effect of *Aronia melanocarpa* fruit juice (AMJ) supplementation on age-related coronary arteries remodeled in aged rat hearts. Male Wistar rats (n = 24) were divided into three groups: (1) young controls (CY), aged 2 months, without AMJ supplementation; (2) old controls (CO), aged 27 months, without AMJ supplementation; and (3) the AMJ group (A), which used 27-month old animals, supplemented orally with AMJ for 105 days. AMJ supplementation did not influence the wall-to-diameter parameter (Kernohan index) of the coronary arteries of test animals. Aged rats supplemented with AMJ showed a significant decrease in the amount of collagen fibers in their coronary tunica media, as compared with the old controls. The intensity of the immunoreaction for alpha smooth muscle actin (αSMA) in the coronary tunica media was significantly lower in the supplemented group than in the old controls. The intensity of the angiotensin-converting enzyme 2 (ACE2) immunoreaction in the coronary tunica media of the supplemented group was significantly higher than the one observed in the old controls. These results indicate the positive effects of AMJ supplementation on the age-dependent remodeling of coronary arteries and support for the preventive potential of antioxidant-rich functional food supplementation in age-related diseases.

## 1. Introduction

The incidence and spread of cardiovascular and cerebrovascular diseases, such as hypertension, coronary heart disease, congestive heart failure, and stroke increase with advancing age. The greater part of research in this field focuses on developing interventions directed to the “traditional” risk factors for coronary heart disease (e.g., hypertension, hypercholesterolemia, etc.), whereas ageing remains outside the focus of attention [1]. With age, the blood vessels undergo structural and functional changes, even in visibly healthy individuals. All three layers of the arterial wall are involved—tunica intima, tunica media, and tunica adventitia. Age-related alterations in the arterial wall and function have been investigated in rodents, rabbits, and primates, and are rather similar to those observed in humans [1,2]. Age-related changes in the cells building up the three layers of the vascular wall underlie the tissue alterations observed. The pathophysiological processes associated with age are triggered by oxidative stress and pro-inflammatory microenvironment mediated by mechanical and humoral factors [3]. The changes in the function and redox status of vascular smooth muscle cells (VSMCs) underlie age-related vascular remodeling. In the process of ageing, the VSMCs alter their phenotype from contractile to a synthesizing one. They cease to interact normally with the extracellular matrix due to the alterations in integrin expression or the alterations in its composition [4,5]. In the ageing process, even when there is no vascular disease present, VSMCs’ migration from the media, and their proliferation, cause intimal thickening in the arterial walls [6]. The aged smooth muscle cells produce a larger quantity of matrix metalloproteinase 9 (MMP 9), thus contributing to the disintegration of extracellular matrix (ECM) [7]. Age-related remodeling of the vascular wall is characterized by the deposition of an excessive amount of collagen, proliferation of VSMCs, accumulation of the extracellular matrix, and suppression of its disintegration. This is a dynamic process, initially occurring as a reversible adaptive reparative response; however, the fibrogenic process progresses, resulting in further deterioration of arterial rigidity. The accumulation of collagenous connective tissue occurs in the large, as well as small, arteries [8]. The combination of ageing and factors favorable for the development of hypertension, as well as the activation of the renin-angiotensin-aldosterone system (RAAS), inflammation, oxidative stress, salt consumption, and genetic factors, result in alterations in delineating the border of pathology [8].

The renin-angiotensin-aldosterone system is a key factor in cardiovascular physiology and pathophysiology. It plays a central role in the structural and mechanical alterations in the vessels. Angiotensin II (Ang II) is the main effect-producing RAAS substance, possessing powerful vasoconstricting, pro-inflammatory, and pro-fibrotic properties [9]. The arterial components of the Ang II-signaling cascade increase with advancing age in rats, non-humanoid primates, and humans [1]. All components of RAAS are present in the cardiomyocytes and the vascular wall. Key elements in the cascade of the RAAS system are the angiotensin-converting enzyme (ACE) and its human homologue, the angiotensin-converting enzyme 2 (ACE2). Apart from being an important regulator of RAAS, ACE2 is a main factor in cardiac function, hypertension, and diabetes [9]. ACE2 has been found to act antagonistically with regard to ACE, so it is the functional mechanism eliminating Ang II and reducing its effects, respectively [10]. Expression of ACE2 is found in the cytoplasm, cell membrane, and cell nucleus of endothelial cells, smooth muscle cells, and cardiomyocytes. ACE2 is involved in regulating the process of proliferation, fibrosis, apoptosis, as well as vascular tone, and endothelial function. The expression of this protein is most marked in embryonic undifferentiated cells, and cells of young individuals, but it decreases with age [11]. Until recently, the processes of age-related remodeling of blood vessels were considered to be definitive and irreversible; however, there is evidence showing that they can be slowed down with the help of medications and non-drug management. The concepts of successful and unsuccessful vascular ageing have been coined, and when no signs have been manifested clinically, the most adequate intervention is the prophylactic non-drug management. Changes to one’s way of life and dietary regimen, are strategies of immense potential.

An ever-increasing amount of evidence has shown that consumption of plant food is associated with a lowered risk of developing arteriosclerosis and diseases associated with oxidative stress [12,13]. Most antioxidants ingested with food are of plant origin. Polyphenolic compounds are found in all plants, and quantitatively, they are the most significant antioxidants taken with food [14]. The fruits of black chokeberry (*Aronia melanocarpa* (Michx). Elliot) are among the richest sources of polyphenols and especially anthocyanins in the plant kingdom [14]. Anthocyanins are known to exhibit a wide range of medicinal properties, such as capillary stabilizing, anti-inflammatory, hypotonic, and collagen stabilizing properties [14]. They play an important role in collagen crosslinking, and they inhibit its degradation by enzymes in the presence of inflammatory processes. In model systems, anthocyanin extracts have shown a cardioprotective effect and an ability to inhibit cancer cell growth [15], and aronia anthocyanins inhibit colon cancer by regulating glutamine metabolism [16]. A number of in vitro and in vivo studies have demonstrated a wide range of applications of black chokeberry juice, extracts, and functional drinks, because of their anti-inflammatory, antimutagenic, anticarcinogenic, lipid lowering, antidiabetic, antihypertensive action, as well as hepatoprotective and immunomodulating effects [14,17,18,19,20]. Evidence of the effect of black chokeberry supplementation on the processes of age-related remodeling of the vascular wall is scarce in the available literature; therefore, the aim of the present study was to determine the effect of *Aronia melanocarpa* juice (AMJ) on age-related coronary artery remodeling in the hearts of aged rats.

## 2. Materials and Methods

### 2.1. Aronia melanocarpa Juice

*Aronia melanocarpa* berries were supplied by the licensed farmer Todor Petkov (Kazanlak, Stara Zagora district, Bulgaria) in the stage of full maturity. The fresh berries were placed in polyethylene bags, frozen immediately and stored at −18 °C until juice extraction. *Aronia melanocarpa* juice (dry solids content 18.8 °Bx) was obtained as described in Daskalova et al. [21] Briefly, five kilograms of frozen fruit were defrosted at room temperature and homogenized in a laboratory blender. The homogenate was transferred into a brown-glass bottle and incubated in a thermostatic shaking water bath at 60 °C for 1 h. After that, the pulp was filtered through a cheesecloth and the liquid fraction obtained was centrifuged and used for the study. The total polyphenol content and the major phenolic components, determined by high performance liquid chromatography (HPLC) of the juice, are shown in Table 1. HPLC analysis were preformed according to [21,22] as follows:

#### 2.1.1. HPLC Analysis of Phenolic Compounds

HPLC analysis of phenolic components was performed on the HPLC system Agilent 1220 (Agilent Technology, Santa Clara, CA, USA), with a binary pump and UV-Vis detector (Agilent Technology, Santa Clara, CA, USA). Separation was performed on the Agilent TC-C18 column (5 μm, 4.6 mm × 250 mm) at 25 °C, and a wavelength of 280 nm was used. The following mobile phases were used: 0.5% acetic acid (A) and 100% acetonitrile (B) at a flow rate 0.8 mL/min. The gradient elution started with 14% B, between 6 min and 30 min, which was linearly increased to 25% B, then to 50% B at 40 min. Results were expressed as mg/100 g FW or per liter juice or nectar.

#### 2.1.2. HPLC Determination of Anthocyanins

Anthocyanins were determined using the HPLC system Agilent 1220 (Agilent Technology, Palo Alto, CA, USA), with a binary pump and UV-Vis detector (Agilent Technology, Palo Alto, CA, USA). A wavelength of 520 nm was used. Anthocyanins were separated using an Agilent TC-C18 column (5 μm, 4.6 mm × 250 mm) at 25 °C. The following mobile phases were used: 5% formic acid (A) and 100% methanol (B) at a flow rate of 1.0 mL/min. The gradient condition started with 15% B, and linearly increased to 30% B at 20 min. Results were expressed as mg/100 g FW or per liter juice or nectar.

### 2.2. Animals

The study included 24 male Wistar rats provided by the Vivarium of Medical University-Plovdiv where they were maintained under standard laboratory conditions (housed in polypropylene cages in a controlled clean air environment at a temperature of 22 ± 3 °C, a 12-h light/dark cycle, and relative humidity of 60 ± 5%). Taking into account the hormonal influences in females, we used only male rats. The rats were divided into 3 groups: (1) young controls (CY), aged 2 months, with no AMJ supplementation; (2) old controls (CO), aged 27 months, with no AMJ supplementation; and (3) AMJ A group (A), which used 27-month old animals, supplemented orally with AMJ (10 mL∙kg^−1^) using drinking water for 105 days. The duration of supplementation was based on our previous studies [21]. Rats were on a standard rodent chow (containing 13.45% protein, 51.6% carbohydrate, 3.40% fat) and tap water ad libitum. The animals of the third experimental group (A) received chokeberry juice diluted 1:1 in the drinking water. The daily dose of juice was calculated for every animal after body weight measurement (twice a month). The animals received clear water after ingesting the daily dose of diluted juice. For the whole experimental period, every animal consumed approx. 440 mL fruit juice. At the end of the experimental period, the animals were anesthetized with i.m. Ketamin/Xilazine (90 mg/kg/10 mg/kg) and euthanized by cervical decapitation. Body weight, body mass index (body weight (g)/nasoanal length^2^ (cm^2^), heart weight, and the heart weight index of the rats of all groups were measured and calculated. The hearts of the animals were fixed in 10% neutral formalin and paraffin-embedded, after which histochemical, immunohistochemical, morphometric, and statistical analyses were performed. The experimental protocol was approved by the Committee on Ethical Treatment of Animals of the Bulgarian Agency for Food Safety (No. 193/2018). All animals were treated in compliance with the Principles of Laboratory Animal Care formulated by the National Society for Medical Research and the Guide for the Care and Use of Laboratory Animals prepared by the National Institute of Health (NIH publication No. 86–23, revised 1996).

### 2.3. Light Microscopy

After washing with a physiological serum, the hearts were cut immediately below the coronary sulcus. After that they were fixed with 10% neutral formalin. After conventional paraffin wax embedding, 5 μm serial sections were cut in order to observe the collagen fibers and smooth muscle cells. The sections were stained with azan (according to Heidenhain; the collagen fibers were stained blue, and the smooth muscle cells were stained red). All investigations were performed on sections of the left ventricle of heart.

### 2.4. Immunohistochemistry

The sections (5 µm-thick) obtained from rat heart were deparaffinized, then subjected to the following procedures: detection of antigenic epitopes with citrate buffer, blocking endogenous peroxidase with 3% hydrogen peroxide, blocking endogenous biotin using a kit (ref: № BBK 120, Scy Tek, Lab. Inc., Logan, UT, USA), blocking non-specific binding using a reagent (Superblock, Scy Tek, Lab. Inc., Logan, UT, USA), followed by incubation for 24 h (at 4 °C) with ACE2 polyclonal antibody 1:200 (E-AB-12224, Elabscience Biotechnology Inc., Houston, TX, USA), after which a second 10 min incubation followed, with a biotinylated secondary antibody (№ AGL015 Scy Tek Lab. Inc., Logan, UT, USA). The reaction was visualized by 3,3′-diaminobenzidine tetrachloride (DAB, Scy Tek Lab. Inc., Logan, UT, USA), and the slices were counterstained with Mayer’s hematoxylin. The same protocol was used for the immunohistochemical analysis of αSMA monoclonal antibody 1:5000 (A-2547, Sigma Chemical, St. Louis, MO, USA). All microphotographs were taken using a Nikon Microphot SA microscope (Olimpus, Japan), combined with Camedia-5050Z digital camera (Olympus, Japan).

### 2.5. Morphometric Analysis

The morphometric analysis involved tissue slices 5μm in thickness, obtained from rat hearts immediately below the coronary sulcus. Quantification of collagen was performed on the azan-stained slices by measuring the percentage of collagen fiber distribution in the tunica media of coronary arteries. The Kernohan index was calculated by measuring in micrometers the wall thickness and the diameter of all cross-sections of coronary arteries (muscular type) present in the slice. Every parameter was measured at least three times for each vessel. The intensity of the immune reaction in the coronary tunica media was measured in arbitrary units (AU) on the slices immunostained for αSMA and ACE2. Using a software, the average intensity of pixels was recorded in arbitrary units in the range 0–256 on microphotographs of the blood vessels, 0 being black, and 256 being white. A minimum of 50 points were measured in the tunica media of each blood vessel at magnification ×400. All measurements involved five slices per animal and an examination of all cross-sections of the coronary arteries present. The measurements were performed using the DP–Soft ver. 3.2 software, Olympus, Japan.

### 2.6. Statistical Analysis

The intergroup comparison was made by means of one-way ANOVA followed by Tukey’s test. The differences were considered significant at *p* < 0.05. The data were processed by SPSS software (version 17.0) and presented as means ± SEM.

## 3. Results

### 3.1. Somatometric Parameters

Table 2 represents the results of the somatometric parameters of the groups studied. Our data showed that black chokeberry juice supplementation did not significantly affect the somatometric parameters and the body mass index.

### 3.2. Chemical Composition of AMJ

As is evident from Table 1, the black chokeberry juice used is a very rich source of several classes of phenolic compounds–anthocyanins, hydroxycinnamic acids, and flavonols. Anthocyanins are represented by Cyanidin-3-galactoside, Cyanidin-3-glucoside, Cyanidin-3-arabinoside, and Cyanidin-3-xyloside, and their cumulative content in the juice exceeded 2100 mg/L.

### 3.3. AMJ Supplementation Did Not Influence Kernohan Index

**Figure 1:**
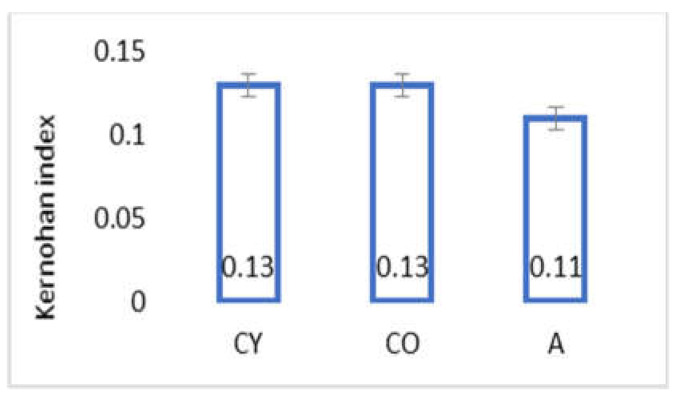
Kernohan index of coronary arteries of the studied groups.

Figure 1 shows the results obtained for the Kernohan index of coronary arteries, calculated on the basis of the data from the morphometric analysis. AMJ supplementation did not influence wall-to-diameter parameter (Kernohan index) of coronary arteries of aged rats, and no significant differences were observed between young and old controls.

### 3.4. Effect of AMJ Supplementation on the Amount of Connective Tissue

**Figure 2:**
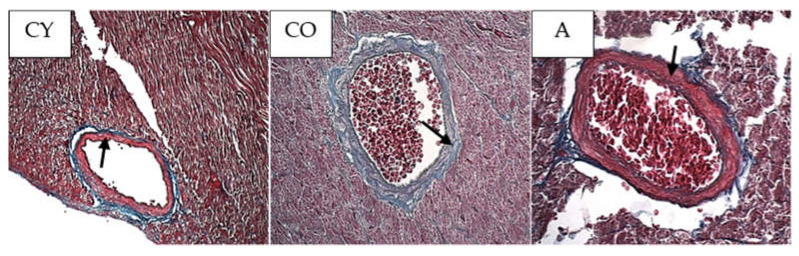
Myocardium. Coronary arteries of the left ventricle of the rat heart, azan staining, magnification × 200; black arrows indicate tunica media.

Figure 2 shows the photomicrographs of the azan-stained slices of rat heart. The blue collagen fibers in the tunica media of the coronary arteries can be visually distinguished and quantified owing to the histochemical technique used.

**Figure 3:**
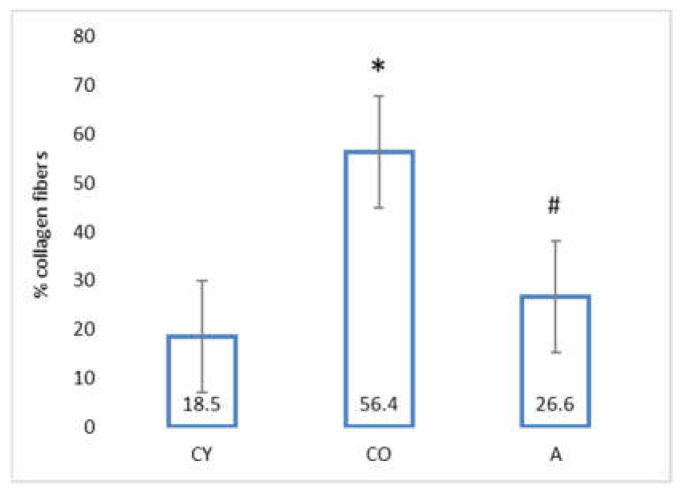
Percentage of collagen fiber distribution in the coronary tunica media of rat heart. * CO vs. CY, *p* < 0.05, # A vs. CO, *p* < 0.05.

The data from the morphometric analysis of collagen fiber distribution in the coronary tunica media are presented in Figure 3.

The amount of collagen fibers in the coronary tunica media of old controls (CO) was significantly increased, as compared with that of young controls (CY), which could be attributed to the natural ageing process; however, supplementation with AMJ resulted in a significant decrease in the amount of collagen fibers in the tunica media of the aronia-supplemented group (A) in comparison with the old controls (CO).

### 3.5. Effect of AMJ Supplementation on αSMA Immunoexpression

**Figure 4:**
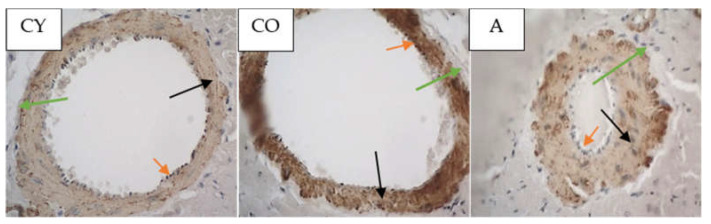
Myocardium. Coronary arteries of left ventricle of rat heart, αSMA immunoreaction in tunica media, magnification ×400. Orange arrows indicate tunica intima, black arrows—tunica media, green arrows—tunica adventitia.

Figure 4 shows the photomicrographs of the αSMA immunoreaction in the tunica media of coronary arteries of rat hearts.

**Figure 5:**
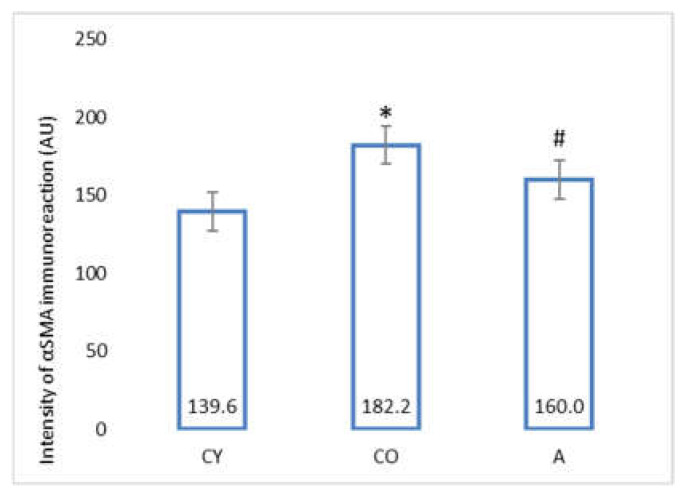
Intensity of αSMA immunoreaction (in arbitrary units) in tunica media of coronary arteries of rat heart. * CO vs. CY, *p* < 0.05, # A vs. CO, *p* < 0.05.

The data from the morphometric analysis of the intensity of αSMA immunoreaction in tunica media of coronary arteries of rat heart are presented in Figure 5.

The old control (CO) group showed a significantly higher intensity of the αSMA immunoreaction, as compared with the young controls (CY), *p* < 0.05, which could be attributed to the natural ageing process. The immunohistochemical reaction for αSMA in the coronaries was significantly intensified in the supplemented animals (A), as compared with the old controls (CO), *p* < 0.05.

### 3.6. Effect of AMJ Supplementation on ACE2 Immunoexpression

**Figure 6:**
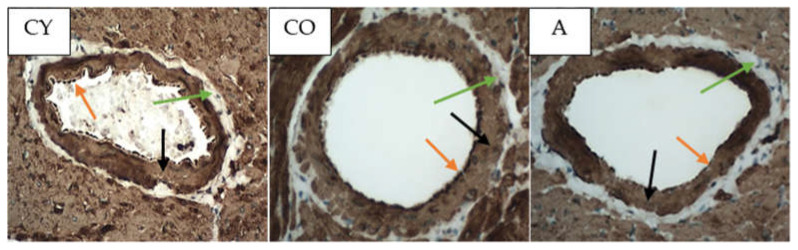
Myocardium. Coronary arteries of left ventricle of rat heart, ACE2 immunoexpression, magnification ×400. Orange arrows indicate tunica intima, black arrows—tunica media, green arrows—tunica adventitia.

**Figure 7:**
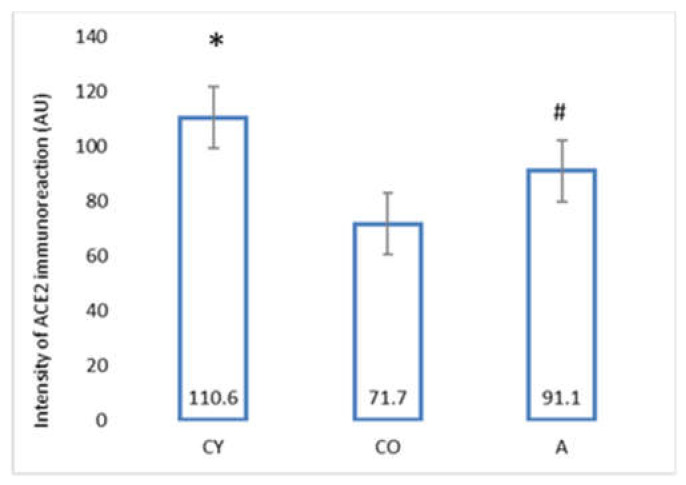
Intensity of ACE2 immunoreaction (in arbitrary units) in tunica media of coronary arteries of rat heart. * CO vs. CY, *p* < 0.05, # A vs. CO, *p* < 0.05.

Figure 6 shows the photomicrographs of the ACE2 immunoreaction in the tunica media of coronary arteries of rat hearts, whereas Figure 7 presents the data from the morphometric analysis of the immunoreaction intensity for ACE2 in the coronary tunica media.

In comparison with the young controls (CY), the intensity of the ACE2 immunoreaction of the old controls (CO) was significantly lower (*p* < 0.05), which could be attributed to the natural ageing process. The morphometric analysis revealed that the intensity of the ACE2 immunoreaction in the tunica media of the supplemented group (A) was significantly increased, as compared with the one observed in the group of old controls (CO), *p* < 0.05.

## 4. Discussion

Body weight and body mass index change significantly in the process of ageing due to the increased adipose tissue in the body on one hand, and changes in lean mass, on the other [23,24]; however, our results showed that aronia juice supplementation did not significantly affect the somatometric parameters and body mass index, which are the criteria for normal body structure. In our study, the Kernohan index remained unchanged in all experimental groups—a fact that indicates a lack of hypertrophy or any other vascular wall pathology and confirms the model of physiological ageing. The wall of the coronary arteries of the aged animals had an unaltered thickness, in spite of the reorganization of the vascular structural elements, which had occurred with ageing. This has been described as eutrophic remodeling [25]. The results obtained from the comparison of young and old controls in our investigation of the amount of collagenous connective tissue (CT) in the tunica media confirmed the results of other authors [8]. Our results showed that the amount of CT in the vascular wall of the aged animals from the supplemented group was reduced, as a result of which, the wall acquired a phenotype characteristic of young animals. Similar results—CT reduction following administration of *Aronia melanocarpa*—have been obtained in other investigations of ours involving the aorta and thymus of adult rats, a fact which supports the results of the present study [26,27]. αSMA is a marker for smooth muscle cells and myofibroblasts in the vascular wall [28,29,30]. A significant increase in αSMA intensity was found in the old controls, as compared with the young controls (*p* < 0.05), which is likely to be a manifestation of the increased number of activated myofibroblasts in the tunica media. This finding also correlates with the increased amount of collagen fibers in the tunica media when comparing these two experimental groups. The myofibroblast transformation of VSMCs is a physiological process of gradual transformation of the contractile phenotype of these cells into a synthetic one under the influence of local and general factors [28]. AMJ administration resulted in a significant reduction of αSMA expression in the supplemented animals, as compared with the old controls (*p* < 0.05). This is a morphological manifestation of slowing down of the ageing process, probably associated with reduced smooth muscle cell activity in the tunica media. The result is a suppressed αSMA expression, with a subsequent reduction of collagenous connective tissue in the vascular wall. The increased collagen synthesis in the vascular wall correlates with the transition of smooth muscle cells in the tunica media from a contractile to a synthetic phenotype. This phenomenon has been described as a feature of ageing smooth muscle cells [4,5].

Harikrishnan et al. conducted a study in which they revealed that αSMA plays a part, not known to scientists up to that moment, in the regulation of type I collagen expression in cardiac fibroblasts treated with Ang II [29]. The increased collagen synthesis correlates with the transition of myocardial fibroblasts to an αSMA-positive phenotype. The expression of αSMA is a sign of the differentiation of myofibroblasts toward a synthetic phenotype. These results clarify the relation between the increased amount of type I collagen and the phenotype transformation of cardiac fibroblasts into producing myofibroblasts [29].

The comparison between young and aged animals revealed a reduced intensity of the ACE2 immunoreaction in the aged animals, a fact that has been found by other authors as well [9,31]. We observed two findings, which parallel the manifestation of the anti-ageing properties of black chokeberry juice. The significantly reduced amount of connective tissue and the increased ACE2 immune expression in the tunica media of the supplemented animals are indicative of a possible influence on the RAAS system. In another study of ours, we found a similar increase in ACE2 immunoexpression in cardiomyocytes, parallel to a reduced amount of collagen fibers in the myocardial interstitium, following AMJ supplementation in aged rats (unpublished data). Activation of RAAS, endothelin, and RAGE (receptor for advanced glycation end products) are different ways that organisms have at their disposal to react to chronic stress. According to Lakatta, the activation of these signaling cascades brings about a chronic inflammatory response, which, on its part, generates an additional oxidative stress contributing to the progression of age-related structural and functional arterial remodeling [31].

Ang II is one of the factors influencing the phenotype change of the VSMCs from a contractile type to a synthetic one [32]. Ang II is not only a powerful vasoconstrictor, but also a pro-inflammatory molecule stimulating cell growth and matrix deposition during arterial remodeling [25]. According to Heeneman et al. ACE and Ang II take part in the remodeling of large and small arteries in hypertension [33]. ACE2 antagonizes the vasoactive and proliferative action of Ang II. Our investigation showed that black chokeberry juice is likely to counteract Ang II pro-inflammatory effects, which is proven by the significantly increased intensity of the ACE2 immunoreaction in the wall of the coronary arteries. The polyphenol resveratrol increases ACE2 expression and decreases the profibrotic protein expression in Ang II-stimulated vascular smooth muscle cells [34]. The favorable effects of polyphenols are attributed mainly to their antioxidant capacity and ability to modulate the cellular antioxidant defense mechanisms by inducing synthesis of detoxication enzymes, such as superoxide dismutase (SOD), catalase (CAT), glutathione S-transferase (GST), glutathione peroxidase (GPx), NAD(P)H: quinone oxidoreductase 1 (NQO1), side by side with others [35,36]. Apart from that, recent studies have provided evidence of the effect of polyphenols as modulators of signaling pathways, such as TNFα, IL-12p40 (a component of the cytokines interleukin IL-12 and IL-23), and p38 MAPK (a class of mitogen-activated protein kinases) [37,38].

Scientific interest in black chokeberry juice has grown significantly in the recent years. The mechanisms of its anti-aging effects have already been described in detail [39,40,41]. The review of Kasprzak-Drozd, (2021) summarizes the evidence of the efficacy of black chokeberry juice and lists the mechanisms of its action with regard to cardiovascular diseases. Examples of these mechanisms are increasing the activity of endothelial nitric oxide synthase (eNOS), glutathione peroxidase (GSHPx), nuclear factor kappa-light-chain-enhancer of activated B cells (NF-κB), nitric oxide (NO), prostaglandin E2 (PGE2), and so on. Moreover, it reduces the activity of the angiotensin converting enzyme (ACE), C-reactive protein, intercellular adhesion molecule (ICAM), IL-6, IL-8, IL-10, reactive oxygen species (ROS), substances reacting with thiobarbituric acid (TBARS), tumor necrosis factor-α (TNF-α), and the vascular cell adhesion molecule (VCAM) and so on [40]. In an experimental model of aging in mice, Zhao et.al. found that chokeberry polysaccharides reduced inflammation and oxidative stress in brain tissue by inhibiting the NLRP3 inflammasome by the AMPK/SIRT1/NF-κB signaling pathway. Activation of the key antioxidant enzymes SOD and CAT has been shown, as well as lowering malondialdehyde (MDA) levels in brain tissue. An improved composition of the intestinal flora has also been found, which reveals an interesting new perspective for elucidating the effects of chokeberry [39].

A number of studies including clinical tests, epidemiological data, as well as in vitro and in vivo investigations with animals have found a cause–effect relationship between a diet rich in polyphenols and its favorable impact on health [42,43]. At present, polyphenols are considered potential therapeutic agents possessing antioxidant properties, which can be used in the management of cardiovascular diseases [44,45,46,47]. Due to their treatment efficacy, they have been designated as anti-ageing molecules [48]. The greater part of traditional medications is directed to act upon the negative health consequences of vascular ageing, rather than its pathophysiology. For instance, the classical antihypertensive therapies reduce peripheral vascular resistance and influence only part of the arterial remodeling directly associated with ageing [25]. The results obtained in the current study are informative, but they cannot be generalized, since the investigation had several limitations. It involves only experimental animals and the study was performed on a tissue level without being supported by serum analyses. Furthermore, only morphological and no physiological indices were studied, and the indices studied provided indirect information on influencing inflammation and oxidative stress. Nevertheless, these results give us grounds to suppose that non-drug agents can influence age-related vascular remodeling, both as primary prevention (prior to the occurrence of pathology) and as secondary prevention (following the occurrence of pathological alterations). This is one of the possible ways to ensure successful vascular ageing. That is why it is justified to study in greater detail the mechanisms underlying the effect of black chokeberry juice supplementation on vascular structures.

Our results provoke interest in another way as well. At present, ACE2 is the focus of attention because of its role in the process of SARS-CoV-2 penetrating and infecting cells. Little is known about the effect of the binding of SARS-CoV-2 virus to ACE2, and the way in which this binding can modulate ACE2 enzyme activity [49]. The experimental data of Bartova et al. [11] suggest that the higher levels of ACE2 in the tissues provide a barrier against SARS-CoV-2 infection.

## 5. Conclusions

The current study demonstrates that supplementation with black chokeberry fruit juice reduces the CT amount and αSMA expression, and increases ACE2 expression in the tunica media of the coronary arteries of aged rats. The positive impact of age-related coronary artery remodeling in rats is a new aspect of the anti-aging action of black chokeberry juice that provides indirect evidence for its anti-inflammatory and antioxidant properties. Even though the obtained results are convincing, our study would be stronger if the morphological tests had been supported by serum and physiological tests. It should be noted that conducting animal experiments have their drawbacks and limitations, but are still indicative enough to support the use of black chokeberry products in humans. Our data support the preventive potential of antioxidant-rich foods in age-related diseases; therefore, *Aronia melanicarpa* fruit juice may prove to be a means for successful vascular ageing.

## Figures and Tables

**Table 1:** Content of anthocyanins and other phenolic compounds in *A. melanocarpa* fruit juice [21].

Total Polyphenols (mg/L)	11,237.4 ± 456.2
Flavonoids (mg/L)	
Quercetin	49.6 ± 3.2
Quercetin-3-β-glucoside	228.8 ± 11.0
Rutin	446.5 ± 12.5
Epicatechin	408.2 ± 25.6
Anthocyanins (mg/L)	
Cyanidin-3-galactoside	1498.4 ± 102.3
Cyanidin-3-glucoside	120.1 ± 8.7
Cyanidin-3-arabinoside	501.9 ± 31.8
Cyanidin-3-xyloside	4.59 ± 0.2
Phenolic acids, mg/L	
Chlorogenic acid	1375.6 ± 80.3
Neochlorogenic acid	1543.1 ± 111.2

**Table 2:** Somatometric parameters of test animals.

	Body Weight (g)	*p* < 0.05	BMI	*p* < 0.05	Heart Weight (g)	*p* < 0.05	Heart Weight Index
CY	158.57		0.52		0.50		0.31
CO	402.14	* CY	0.70	* CY	1.06	* CY	0.26
A	417.86	# CY	0.69	* CY	1.06	# CY	0.25

Body weight (g) * CO vs. CY, *p* < 0.05; # A vs. CY, *p* < 0.05; Body mass index * CO vs. CY, *p* < 0.05; # A vs. CY, *p* < 0.05. Heart weight (g) * CO vs. CY, *p* < 0.05; # A vs. CY, *p* < 0.05.

## Data Availability

The data presented in this study are available on request from the corresponding author.
